# Contextualizing and pilot testing the Mental Health Gap Action Programme Intervention Guide (mhGAP-IG) to primary healthcare workers in Kilifi, Kenya

**DOI:** 10.1017/gmh.2020.6

**Published:** 2020-05-18

**Authors:** Mary A. Bitta, Symon M. Kariuki, Anisa Omar, Leonard Nasoro, Monica Njeri, Cyprian Kiambu, Linnet Ongeri, Charles R. J. C. Newton

**Affiliations:** 1 Clinical Research-Neurosciences, KEMRI/Wellcome Trust Research Programme, Centre for Geographic Medicine Research (Coast), Kilifi, Kenya; 2 Department of Psychiatry, University of Oxford, Oxford, UK; 3 Department of Health, County Government of Kilifi, Kilifi, Kenya; 4 Centre for Clinical Research, Kenya Medical Research Institute, Nairobi, Kenya

**Keywords:** Interventions, Kenya, mental disorders, mhGAP, neurological disorders

## Abstract

**Background:**

Little data exists about the methodology of contextualizing version two of the Mental Health Gap Action Programme Intervention Guide (mhGAP-IG) in resource-poor settings. This paper describes the contextualisation and pilot testing of the guide in Kilifi, Kenya.

**Methods:**

Contextualisation was conducted as a collaboration between the KEMRI-Wellcome Trust Research Programme (KWTRP) and Kilifi County Government's Department of Health (KCGH) between 2016 and 2018. It adapted a mixed-method design and involved a situational analysis, stakeholder engagement, local adaptation and pilot testing of the adapted guide. Qualitative data were analysed using content analysis to identify key facilitators and barriers to the implementation process. Pre- and post-training scores of the adapted guide were compared using the Wilcoxon signed-rank test.

**Results:**

Human resource for mental health in Kilifi is strained with limited infrastructure and outdated legislation. Barriers to implementation included few specialists for referral, unreliable drug supply, difficulty in translating the guide to Kiswahili language, lack of clarity of the roles of KWTRP and KCGH in the implementation process and the unwillingness of the biomedical practitioners to collaborate with traditional health practitioners to enhance referrals to hospital. In the adaptation process, stakeholders recommended the exclusion of child and adolescent mental and behavioural problems, as well as dementia modules from the final version of the guide. Pilot testing of the adapted guide showed a significant improvement in the post-training scores: 66.3% (95% CI 62.4–70.8) *v.* 76.6% (95% CI 71.6–79.2) (*p* < 0.001).

**Conclusion:**

The adapted mhGAP-IG version two can be used across coastal Kenya to train primary healthcare providers. However, successful implementation in Kilifi will require a review of new evidence on the burden of disease, improvements in the mental health system and sustained dialogue among stakeholders.

## Background

The mental health Gap Action Programme (mhGAP) was first published by the World Health Organization (WHO) in 2008, in response to the recognition of a need to scale up delivery of mental health services by utilising available resources within a health system (WHO, 2008). The mhGAP Intervention Guide (mhGAP-IG) for mental neurological and substance use disorders (MNSD) is a protocol for clinical decision making that was developed through a systematic review of evidence and international consultations to aid in the implementation of the mhGAP (WHO, 2015*a*). Since its inception, over 90 countries have taken up its use, although literature is dominated by only a few countries as reported in a systematic review on the implementation of the mhGAP (Keynejad *et al*., 2018). This review also indicated that the largest proportion of studies (46%) used the guide for training courses, while other uses included clinical implementations, formation of economic models and as rating scales. From this review, only three studies reported local adaptation of the guide and of these, only one study in Nigeria provided a step by step account of the mhGAP-IG contextualisation (Abdulmalik *et al*., 2013). Some of the lessons learnt from Nigeria that informed contextualisation of the guide to their setting included the large gaps in the training curriculum of primary healthcare workers and the importance of establishing a clear referral pathway with proper clinical supervision for the trained health workers. However, this study contextualised version one of the guide (WHO, 2010) which have since been revised (WHO, 2016*a*). More recently, an evaluation of the implementation of the revised guide in Fiji reported that although the guide were deemed valuable and easy to use by the healthcare providers, there was need to improve planning and leadership in the implementation (Charlson *et al*., 2019). However, data on adaptation of the revised guide from low- and middle-income countries are lacking. Lack of data on local adaptation of the revised guide creates a missed opportunity for exploring the real-world challenges of implementing the guide such as language barriers and poor infrastructure which may explain the persistently large treatment gaps (Fekadu *et al*., 2019) in countries that have successfully implemented these guide.

In Kenya, version 1 of the mhGAP-IG has been contextualised (Mutiso *et al*., 2018), and used for clinical practice in both rural and urban areas of eastern Kenya with good specificity (86%), but low sensitivity (46%) (Musyimi *et al*., 2017). The low sensitivity may be attributed to lack of recognition of some disorders by local communities (Bitta *et al*., 2019). However, there are no studies on adaptation and implementation of version two of the guide mhGAP-IG (mhGAP-IG V2). The present study adapted and pilot tested guide mhGAP-IG V2 in coastal Kenya. The pilot testing was conducted among primary healthcare providers to assess the ease of use of the guide and the changes in level of knowledge after training.

## Methods

The contextualisation process was initiated in 2016 by the Kenya Medical Research Institute- Wellcome Trust Programme (KWTRP) in collaboration with the Department of Health of the County Government of Kilifi. The KWTRP is a research institute, formed in 1989 as a collaboration between the Kenya Medical Research Institute, Wellcome Trust and Oxford University from the UK. The institute conducts medical research of both communicable and non-communicable diseases in an established demographic and surveillance system (Scott *et al*., 2012). The department of health in Kilifi was established in 2010 after the implementation of the devolved system of government in Kenya, which saw the establishment of 47 independent administrative units called counties, including Kilifi (KIlifi, 2019). Permission to conduct this study was obtained from the Scientific Ethics and Review Unit of the Kenya Medical Research Institute under protocol number 038/3260. The steps of the contextualisation process are described below.

### Step I: situation analysis of the state of mental health services in Kilifi Kenya

To understand the Kenyan context, we conducted a narrative synthesis of publicly available data. Our data sources included: (i) the Ministry of Health, where we obtained data on the structure of the formal health system, expenditure on health and human resource for health (GOK, 2014, 2015); (ii) published peer reviewed articles for data about alternative pathways to mental healthcare in Kenya (Mbwayo *et al*., 2013; Burns and Tomita, 2015; Musyimi *et al*., 2018); (iii) Kenya National Bureau of Statistics for Kenya's demographics (KNBS, 2009); (iv) the Constitution Of Kenya for data about mental health legislation (GOK, 2015) and (v) the Mental Health Atlas and published literature for data about the burden of mental illnesses in Kenya (Jenkins *et al*., 2012; Kariuki *et al*., 2015; WHO, 2015*b*; Otiende *et al*., 2017; Bitta *et al*., 2018). We then conducted a situational analysis of the state of mental health systems in Kilifi using the brief version of the World Health Organization's Assessment Instrument for Mental Health Systems (WHO-AIMS) (WHO, 2009). This instrument quantitatively assesses a mental health system based on six main domains: (i) policy and legislative framework; (ii) mental health services; (iii) mental health in primary care; (iv) human resources; (v) public education and links with other sectors; and (vi) monitoring and research. Data were either binary (i.e ’yes or “no’) or numerical (e.g. the number of staffs, or the year a policy was developed etc). The data were entered in a standardised WHO- AIMS 2.2 Excel spreadsheet. Descriptive statistics were conducted using Stata version 13 (Stata Corp, Texas, USA) and a binomial distribution was used to generate confidence intervals.

### Step II: strategic stakeholders meeting to promote involvement

The aim of these meetings was to discuss the feasibility of implementing the mhGAP-IG in Kilifi County. The meetings, which lasted about an hour, were conducted with the National Director of the Department of Mental Health Substance Use, County Director of Health and County Head of Community Strategy. These stakeholders were identified through consultation with KWTRP's community liaison team as well as through previous engagements between KWTRP's researchers and the County government. At both the county and national level, we asked the stakeholders for potential facilitators and barriers to the successful implementation of the mhGAP-IG in Kilifi. Additionally, we asked the County level stakeholders about their anticipated roles and those of the research institute (KWTRP) in implementation. These were informal introductory meetings and consent was not obtained to record the conversations or to quote the findings verbatim, but we obtained oral consent to take notes during the meetings and analyse the findings. We used a constant comparison approach with content analysis to summarise the key findings of the discussions.

### Step III: focus group discussions with primary healthcare providers, traditional health practitioners and expert meetings to contextualise the guide

Focus group discussions were conducted with primary healthcare providers and traditional health practitioners to understand the local terms, presentations and management of the priority MNSD in Kilifi. The discussions aimed to provide a qualitative situational analysis of the understanding and management of the disorders in Kilifi. This would inform adaptation of the mhGAP-IG training materials, (e.g. to make role plays and examples used during trainings locally relevant). Traditional health practitioners were included because previous studies in Kilifi (Kendall-Taylor *et al*., 2009) show that they are an integral part of the pathways to healthcare among people with the disorders in Kilifi. Purposive sampling was used to identify primary healthcare providers who were selected to ensure maximum variations in age, sex and geographical locations. Traditional health practitioners were identified through a database established in a previous study (Kendall-Taylor *et al*., 2008). Informed consent was obtained from all participants. Using vignettes which were adapted from a primary healthcare providers' mental health services handbook (Patel, 2018) to represent each of the priority conditions, an experienced moderator who was fluent in both English, Kiswahili and Kigiriama (the local language) asked participants the following questions: (i) What are the local terms for these disorders? (ii) How do people with these disorders present in the community? (iii) How do you manage these disorders? An example of a vignette for psychosis is provided in Table 1. The questions were used as a framework for content analysis where two investigators independently coded the data using an inductive approach and then compared and discussed their findings until consensus was reached.

**Table 1.** An example of a vignette used for psychosis

The cases were in the form of vignettes adapted from primary healthcare providers mental health services handbook (Patel, 2018). An example of a vignette for the psychosis module was as follows: *“Gambo, a 20-year-old university student, was brought* [to traditional healer/primary healthcare provider] *because she locks herself in her room. Gambo used to be a good student but has failed her last exams. Her mother said that she would often spend hours staring into space. Sometimes she appeared to say things to herself in a low and inaudible voice as if she were talking to an imaginary person. Gambo was forced to come to the clinic/traditional healer by her parents. At first, she refused to talk to the nurse/ traditional healer. After a while she admitted that she believed her parents and neighbours were plotting to kill her and that the devil was interfering with her mind. She also said that she did not see why she had been brought to the hospital/traditional healer”.*

To contextualise the guide, the mhGAP-IG contextualisation questionnaire (Abdulmalik *et al*., 2013) was used by specialists who included three psychiatric nurses, a clinical psychologist, two epilepsy specialists and two clinical officers with mental health experience. The master chart, which will be displayed in facilities and will be used as the first assessment tool, was translated to Kiswahili (Appendix 1) which is the lingua franca 7 in Kenya. The specialists reviewed the recommendations of the guide against the availability and levels of skill and competence of the primary healthcare providers who would implement this guide and against the availability of resources at present and their likelihood of being available in the future. Only essential changes were made. These were changes that were unlikely to improve soon or which were not relevant to the setting. For instance, we revised the drugs recommended in the guide to align with those in the County's essential drugs list Table 2.

**Table 2.** Availability of essential medicines in Kilifi County based on Kenya's essential medicines list for mental illnesses

Antipsychotics	Formulation	Dosage	Available in Kilifi County
Chlorpromazine HCl	Injection	(a) 25 mg/ml (2 ml amp)	Yes
	Tablet	(b) 50 mg	No
		(c) 100 mg 4	Yes
Flupentixol decanoate	Injection (oily, depot)	20 mg/ml (2 ml amp)	No
Fluphenazine decanoate	Injection (oily, depot)	25 mg/1 ml amp	Yes
Haloperidol	(a) Injection	5 mg/1 ml amp	Yes
	(b) Oral liquid^a^	2 mg/ml	
	(c) Tablet (scored)	5 mg	Yes
Olanzapine^b^	PFI	10 mg vial	No
Quetiapine	Tablet	200 mg (scored	No
Zuclopenthixol acetate	Injection (oily)	50 mg/ml (2 ml amp)	No
	Injection (depot, oily)^c^	200 mg/1 ml amp	No
Specialist list			
Chlorpromazine HCl	Injection	25 mg/ml (2 ml amp)	Yes
Clozapine	Tablet (scored)	(a) 25 mg	Yes
		(b) 100 mg	Yes
Haloperidol	Injection	(a) 5 mg/1ml amp	No
	Tablet (scored)	5 mg	Yes
Risperidone	Tablet	2 mg (scored)	No
Medicines used in depressive disorders			
Amitriptyline HCl	Tablet	25 mg	Yes
Fluoxetine	Tablet (scored)	20 mg (as HCl)	Yes
Specialist list			
Fluoxetine^d^	Tablet (scored)	20 mg (as HCl)	No
Venlafaxine	Capsule	75 mg (as HCl)	No
Medicines used in bipolar disorders			
Carbamazepine	Tablet	200 mg (cross-scored)	No
Valproic acid (sodium valproate)	Tablet (enteric-coated)	(a) 200 mg	Yes
		(b) 500 mg	No
Specialist list			
Lithium carbonate	Tablet	400 mg (modified release)	No
Medicines used in anxiety disorders			
Bromazepam^e^	Tablet (scored)	3 mg	No
Medicines used in obsessive compulsive disorders			
Clomipramine HCl	Capsule	25 mg	No
Medicine used in disorders due to psychoactive substance abuse			
Diazepam^f^	Tablet	5 mg	Yes
Nicotine	Chewing gum	(a) 2 mg	No
		(b) 4 mg	No
Specialist list^g^			
B vitamins, high potency	Injection IM	7 ml (in 2 amps)^h^	Yes
	Injection IV	10 mL (2 × 5 ml amps)^i^	Yes
Buprenorphine + naloxone (both as HCl)	Tablet (sublingual)	(a) 2 mg + 500 mg	
		(b) 8 mg + 2 mg	
Methadone HCl	Oral liquid	5 mg/ml (concentrate)	No
Naltrexone HCl	Tablet	50 mg	No
Medicine used in attention deficit hyperactivity disorder			
Methylphenidate	Tablet	10 mg	Yes

a Drops with dosing pipette.

b Use only in patients refractory to, or intolerant of, 1st generation antipsychotic.

c Use only in patients refractory to, or unable to tolerate, other antipsychotics.

d Only use in patients >8 years.

e Only use in anxiety with agitation.

f Only use in the management of alcohol dependence.

g Use under close supervision within substance dependency treatment programmes.

h Ascorbic acid 500 mg, nicotinamide 160 mg, pyridoxine hydrochloride 50 mg, riboflavin 4 mg, thiamine hydrochloride 250 mg/7 ml.

i Ascorbic acid 500 mg, nicotinamide 160 mg, pyridoxine hydrochloride 50 mg, riboflavin 4 mg, thiamine hydrochloride 250 mg/10 ml.

### Step IV: pilot testing of adapted guide among primary healthcare providers and post-pilot feedback workshop among specialists as well as primary healthcare providers

The pilot sites were three primary healthcare facilities within Kilifi County. Convenience sampling was used to select participants of the pilot to represent different cadres, age groups and sex. A 1-week training on the use of the guide was facilitated by a total of seven trainers with each trainer facilitating one module in the guide. The mhGAP-IG training manuals (WHO, 2016*b*) guided the training sessions. The training included both theoretical skills of the common signs and symptoms and management modalities of the priority disorders as well as hands on skills such as communication and physical examination skills as specified in the training manuals. For each module, pre and post-training knowledge questionnaires, which are part of the training materials, were administered to the healthcare providers to measure their changes in level of knowledge. The pre- and post-training scores on levels of knowledge were compared using a Wilcoxon signed-rank test. A feedback meeting among the specialists and representatives of the trainees was conducted to review the training.

## Results

### Step I: situation analysis of the state of mental health services in Kilifi Kenya

#### The Kenyan context

Kenya is an East African country with a population of approximately 47.6 million people and about 44 officially recognised tribes although there are about 70 different ethnic groups. Kenya's basic governance system is two tiered and includes a National level, headed by a president and a County level headed by a governor. There are 47 counties in Kenya and each independently sets priorities and operations in terms of revenue generation and resource allocation. The National Government is responsible for national referral facilities and health policy and legislation; while the County government is responsible for human resource for health and infrastructural development and support. Kenya has an essential healthcare package which comprises five levels namely: community level, (dispensaries or clinics) health centres, primary hospitals, secondary hospitals and tertiary facilities. Approximately 60% of all health services are provided by the public health sector while the remainder 40% are provided by private and faith-based sectors. The ratio of healthcare workforce per 100 000 population is 20.4 for doctors, 158.2 for nurses and midwives, and 5.0 for other healthcare providers such as nutritionists, occupational therapists and counsellors. Additionally, there is an informal health sector composed of traditional health practitioners and faith healers mainly in rural areas as well as unlicensed biomedical practitioners mostly based in urban areas. As a result, the pathways to healthcare are tortuous and sometimes shift between formal and informal care, especially for mental and neurological disorders.

Kenya contributes data to the Mental Health Atlas project through the Department of Mental Health and Substance Use. According to these data, in 2017, mental illnesses contributed approximately 2467 disability-adjusted life years (DALY) per 100 000 population and depressive disorders were one of the top ten leading causes of DALY. Mental health infrastructure is poorly developed, and disproportionately distributed in favour of major cities situated in a few counties. There is only one standalone national referral mental specialist hospital in Kenya, 15 psychiatric in-patient units in general hospitals with about 12 000 patients, 97.8% of whom are involuntarily admitted. There are 30 mental health outpatient units, which receive approximately 741 visits per 100 000 population annually. The mental health expenditure per person per year is approximately 0.03 dollars, which is only 0.01% of the total national government expenditure on health. There are 92 psychiatrists, four of whom are child and adolescent mental health specialists. The mental health workforce is about 0.19 per 100 000 population. The Kenya Mental Health Act of 1989 is the sole stand-alone law for mental health in Kenya. It is outdated with sections that are inconsistent with the current international standards such as lack of an independent oversight authority that assess compliance of this legislation with international laws on human rights. Kenya has a mental health policy that was developed in 2015 and includes a mental health plan with specific indicators against which implementation can be evaluated. Key among the priority areas is human resource development and one strategic action relevant to this study is the provision of in-service training for health workers, to build capacity for management of mental illnesses.

#### The Kilifi context

Kilifi County is one of six coastal counties of Kenya, with the others being Mombasa, Kwale, Tana River, Lamu and Taita Taveta. It is a largely rural area on the coast of Kenya and comprises six sub-counties namely, Kilifi, Ganze, Malindi, Magarini, Rabai and Kaloleni and covers a total surface area of 12 609.7 km^2^ (2.1% of the country). The population of the County is estimated to be 1.4 million. The main communities include the Mijikenda, Bajuni, Swa-Arab, Indian, European decent and other migrant Kenyan communities. The main economic activities are agriculture (cashew nuts and horticulture), fishing, forestry and mining. Kilifi County has 156 public health facilities (one teaching and referral hospital, four sub-County hospitals, 14 health centres and 137 dispensaries) and 151 private facilities including a neurology clinic run by the Neuroscience Department of the KEMRI-Wellcome Trust Research Programme in collaboration with Kilifi county Department of Health. This clinic provides free follow-up care mainly for research participants with epilepsy and neurodevelopmental disorders but also for members of the public who are referred from other facilities. There are three public outpatient facilities attached to general hospitals that offer psychiatric outpatient care which manage about 180 psychiatric patients per 100 000 populations based on the number of outpatient visits only. The doctor-patient ratio in Kilifi County is low at 10 per 100 000 population whereas the nurse-patient ratio is 40 per 100 000 population compared to WHO's recommendation of 333 per 100 000 population and 250 per 100 000 population, respectively. Health services are managed by the county Health Services Department, which reports to the national Ministry of Health. The county director of health works closely with the County health management team and provides leadership to the department of health. Kilifi County Hospital serves as the mainstream teaching and referral hospital in the County. It provides both outpatient and inpatient services and has a total bed capacity of 184.

Results of the analysis using WHO-AIMS have been presented extensively elsewhere (Bitta *et al*., 2017). In summary, there were no regional policy and legislative frameworks specifically for mental health, hence prompting the coastal region to use national laws and regulations. Infrastructure for specialised mental healthcare was limited to three psychiatric outpatient units attached to general hospitals. In-patient psychiatric care was provided in general hospitals based on the availability of beds. Based on Kenya's essential drugs list for managing psychiatric conditions, less than half of the drugs were available in Kilifi county as shown in Table 2. The supply of drugs was erratic. Specialised human resource for mental health comprised three psychiatric nurses and one clinical officer for a population of approximately 1.2 million people, giving a provider patient ratio of 1:300 000. There are no efforts to integrate mental health with primary care and monitoring and research in the region is mainly conducted by KWTRP. Community surveys have been conducted for neurological disorders, mainly epilepsy and acute seizures, some mental illnesses such as behavioural and emotional problems in children and suicide as a fatal outcome of mental illness.

### Step II: strategic stakeholders meeting to promote involvement

As we did not obtain consent to record and use verbatim data obtained during these meetings, we do not provide quotations in this section. Although we had originally aimed to reach three key stakeholders, we conducted a total of six meetings over a period of 1 year with the following stakeholders :(i) National Director of the Department of Mental Health and Substance Use; (ii) Chief Officer of Health in Kilifi County; (iii) the County Head of Community Strategy; (iv) the County Coordinator of non-communicable diseases and research and (v) two medical superintendents from Kilifi County Hospital and Malindi Sub-County Hospital. These stakeholders were identified by snowballing from the three key stakeholders that we had initially targeted. With regards to the barriers to successful implementation, the stakeholders identified low levels of human resource at the primary care facilities, lack of skilled personnel to offer supervision to the trained healthcare providers and lack of clarity on the roles of the research institute and the County government. Potential facilitators of successful implementation included the positive 30 year working relationship between the County government of Kilifi and the research institute (KWTRP). Additional findings from the County and national levels and key outputs of these meetings were summarised in Table 3.

**Table 3.** A summary of the stakeholder meetings and key outputs of the meeting

Stakeholder	Number of meetings	Aim of meeting	Method of data collection	Method of data capture	Analytic method	Key finding (s)	Key outputs of the meetings
National government stakeholders	1	Introduce the research team Identify potential facilitators and barriers to implementation	Unstructured/informal discussions [10]	Notes^a^	Thematic content analysis	Human resource constraints within the health system which would compromise the proposed task-sharing approach provided in the mhGAP,	Linkage with psychiatrist in charge of national mental health research activities. The research team would liaise with the psychiatrist to update the national government on the implementation progress.
County director of health and/or their representatives	5	Introduce the mhGAP Discuss feasibility of the implementation and potential work plan implementation plans Define roles and responsibilities of partners in the collaboration Identify potential facilitators and barriers to implementation	Unstructured/informal discussions [12]	Notes	Constant comparison approach with thematic content analysis	Role of KWTRP and the county government's department of health needed clarification. The stakeholders considered the project timely and feasible for this setting. They, however, required assurance that KWTRP would continue to play a facilitatory role in implementing the guidelines as they did not have the skills and expertise to do so. The longstanding positive working relationship between the county government and KWTRP was seen as a facilitator to successful implementation of the guidelines.	Clear description of the roles of the county government and KWTRP in the implementation process. KWTRP would play a facilitatory role and support the trainings and linkage with World Health Organization's mhGAP office while the county government would be responsible for implementation and scale up of the guidelines. Permission to submit a research protocol to the national ethics and review unit to allow for evaluation of the implementation process and of the effectiveness of the guidelines. Linkages with the community health nurses

a Since this was the first meeting, we did not tape-record the conversations as this may have created a communication barrier between the researchers and respondents.

### Step III: focus group discussions with primary healthcare providers, traditional health practitioners and expert meetings to contextualise the guide

In total, eight focus group discussions, four for each group, were conducted at which point information saturation was achieved. In total there were 60 participants and each group had seven or eight participants. There were local terms for all the priority disorders except dementia which was not recognised by either of the groups. Child and adolescent mental and behavioural problems were not recognised as mental illnesses but as consequences of poor parenting. Most of the common presentations listed in the mhGAP were identified by the participants. However, among the traditional health practitioners, names of unrelated conditions were used simultaneously (e.g. tetanus and epilepsy), suggesting potential misdiagnosis. Management for the disorders among traditional health practitioners depended on the perceived cause of illness and on their expertise. It included referral to biomedical facilities in cases where a patient was not responding to traditional treatment. Management among primary healthcare providers ranged from no care at all, to unstructured counselling to medication to alleviate acute symptoms (e.g. diazepam for agitation in a patient with depression). A detailed summary of these findings including quotations to reflect these findings is published elsewhere (Bitta *et al*., 2019). During translation of the master chart, there were no Kiswahili equivalent words for some disorders such as dementia and child and adolescent mental and behavioural disorders. Only the most essential changes were made to the guide as shown in Table 4. The changes included clarifications of the referral pathways to indicate that specialists in the setting referred to psychiatric nurses and clinical officers specialised in psychiatry as well as adaptation of suggested pharmacotherapy to tally with the drugs that are available in the County (e.g. fluoxetine was replaced with amitriptyline).

**Table 4.** Examples of suggested contextualisation of the mhGAP-IG

Page	Question to be considered	Response	Suggested contextualisation of mhGAP Intervention Guide	Suggested contextualisation of training materials used for training on the mhGAP-IG	Reasoning or technical basis for the suggested change Further information needed before deciding on contextualisation
P23 left column bottom	The text states ‘ Are these signs and symptoms suggesting hypothyroidism, anaemia, malnutrition, mood changes from substance use and medication side-effects tumours, Given what is known about the epidemiology in the country, should the examples of diseases be somewhat changed?	YES	Include malaria (cerebral) and diabetes mellitus	−^a^	Malaria is endemic in this region and patients with diabetes mellitus also present with these symptoms
P22 and 31 middle box	The text says ‘consult with a specialist’. What does consult mean? (Phone? refer?) What specialist should be consulted? (A psych nurse? A psychiatrist?)	Specialist in this case means a psychiatric nurse or clinical officer specialised in psychiatry county	Physical referral with evidence of referral	–	–
P 28 middle column middle row	The text states ‘ if symptoms persist or worsen despite psychosocial interventions, consider fluoxetine (but not other SSRIs or TCAs)’ (1) Is fluoxetine available in PRIMARY HEALTHCARE PROVIDERS? (2) Should children adolescents with depression be referred if the first line of treatment (psychosocial) does not work or should they be given fluoxetine?	(1) NO (2) YES	Change to ‘refer to the next level of care’ Refer adolescent rather than give amitriptyline	–	Fluoxetine is not available in this setting, use amitriptyline instead

a There were no suggested changes.

### Step IV: pilot testing of adapted guide among primary healthcare providers and post-pilot feedback workshop among specialists as well as primary healthcare providers

In total, 16 primary healthcare providers were trained. They included one medical officer, two clinical officers and 13 nurses of different cadres. Overall, there was a significant difference in the mean knowledge scores pre-training *v.* post-training scores 47.0% (s.d. = 3.9) *v*. 60.8% (s.d. = 4.9) *p* < 0.001. In the individual modules, there was significant improvement in knowledge for all modules except for the epilepsy and essential care and practice modules as shown in Table 5. The stakeholders recommended exclusion of the child and adolescent and dementia modules. During the feedback workshop, the final version of the guide was approved after including the changes highlighted in Table 4.

**Table 5.** Pre- and post-training scores of the pilot training among primary healthcare providers

Module	Denominator (maximum score)	Pre-test mean scores (s.d)	Post-test mean scores (s.d)	*p* value for paired test
Essential care and practice	15	8.34 (1.77)	8.83 (2.08)	0.369
Substance use disorders	12	8.38 (1.58)	9.88 (1.99)	0.001
Epilepsy	10	8.59 (1.39)	9.14 (0.91)	0.208
Child and adolescent mental health problems	13	8.40 (2.27)	9.2 (1.83)	0.013
Depression	10	8.27 (1.61)	8.88 (1.31)	0.001
Psychosis	9	6.57 (1.21)	7.62 (1.39)	0.000
Suicide	8	4.92 (1.29)	6 (1.24)	0.001
Overall mean score (s.d)	77	46.95	60.76 (4.87)	0.000

## Discussion

The process of contextualising mhGAP-IG V2 in one of the poorest regions of Kenya has shed light on the gaps and opportunities that a primary healthcare system offers in a devolved system of governance. This study also offers unique insight into the process of collaboration between a research programme (KWTRP) and the County government in adopting and implementing the guide. Our study identified strengths such as willingness of primary healthcare providers to participate in the intervention and challenges such as an excessively strained human resource that were previously identified in other settings in Kenya (Mutiso *et al*., 2018). We also identified opportunities and challenges that have not been previously reported in the contextualisation of the mhGAP-IG in Kenya, such as a good pre-existing collaboration between a research institute and a County government. We discuss how lessons learnt from each stage informed contextualisation.

### Situation analysis of the state of mental health services in coastal Kenya

The situational analysis contributed knowledge to the already available evidence that mental health systems in many low-and middle-income countries are poorly resourced (Roberts *et al*., 2014; Luitel *et al*., 2015; Spagnolo *et al*., 2018). This may have direct negative consequences on the implementation efforts of the mhGAP-IG. For instance, even with correct diagnosis, management of MNSD may fail due to erratic supply of drugs which will lower patients' adherence to medication and lead to poor outcomes. Additionally, lack of trained healthcare providers to identify MNSD and experts to manage MNSD significantly disrupts the referral chain recommended in the mhGAP-IG. For this setting, the human resource limitations can be addressed by ensuring that ‘gate keepers’ of health (i.e. healthcare providers at peripheral facilities) are trained on correct diagnosis and management of priority MNSD so that the few available experts can focus on managing complicated cases, rather than primary diagnosis. In the Kenyan system where drug supply is based on demand (Aronovich and Kinzett, 2001) the creation of demand through increasing case detection and drug prescription may increase drug supply.

### Strategic stakeholders meeting to promote involvement

Studies on implementation of interventions (Frieden, 2014; Spiel *et al*., 2018), including the mhGAP-IG (Dos Santos *et al*., 2019) have demonstrated that gaining stakeholder support is not only necessary for successful implementation of interventions, but also for adoption of evidence generated from the intervention into policy and practice. The engagements in this study provided vital linkages to strategic implementation partners. For instance, we obtained crucial linkages to the national government, the county hospitals and the community health teams which facilitated wider participation in the process. Additionally, the devolved system of governance made stakeholder engagement easier, since both health and administrative services were concentrated within a small geographic region. The engagements identified a key challenge unique to this context: there was a lack of clarity on the roles and frameworks of operation between the County government and the KWTRP (which was facilitating this process). This may have arisen as it is the first time KWTRP was facilitating the implementation of a mental health intervention that directly involved County government health staff on a large scale. However, this was resolved after clarification by the KWTRP that they would only play a facilitatory role. The positive longstanding working relationship between KWTRP and Kilifi County officials that saw KWTRP establish a health and demographic surveillance system (Scott *et al*., 2012) may have contributed to collaboration. Although the nature of this collaboration is beyond the scope of this paper, it is important to note that expectations from different partners in the implementation process may influence the outcome of the process. For this setting, before implementation, there will be need for further engagement with the stakeholders through theory of change workshops (De Silva *et al*., 2014) to address barriers that may hinder task sharing such as competing responsibilities for management of other illnesses and low human resource.

### Focus group discussions with primary healthcare providers, traditional health practitioners and expert meetings to contextualise the guide

Most priority disorders were recognised by both traditional health practitioners and primary healthcare provider. However, dementia and child and adolescent mental and behavioural problems were not regarded as priority conditions by primary healthcare providers and they were not recognised by traditional health practitioners. Lack of recognition of these disorders may be due to lack of knowledge about these illnesses rather than due to a low burden of disease as shown by a 13% prevalence of behavioural and emotional problems in children in Kilifi (Kariuki *et al*., 2017). Exclusion of this module by countries that have implemented the guide including South Africa, Nepal and Uganda (Lund *et al*., 2016) created a missed opportunity of evaluating the barriers and facilitators of successful implementation, especially in sub-Saharan Africa.

Inclusion of traditional health practitioners in the contextualisation process promoted the process of referral because evidence from this setting suggested that traditional health practitioners are an integral part of the healthcare system (Kendall-Taylor *et al*., 2008). The unidirectional willingness of the traditional health practitioners to cooperate with biomedical practitioners has been reported in other parts of sub-Saharan Africa (Akol *et al*., 2018). Traditional health practitioners play a critical role in the referral pathway and can offer psychosocial support. It is critical to address the underlying causes of the strained relationship with the biomedical practitioners, throughout the implementation process.

During the translation of the master chart, which is part of the guide, there were no equivalent Kiswahili words for most of the mental and neurological illnesses. This resulted in the use of phrases which lengthened the guide. The delicate balance between a concise guideline and fidelity of the translations highlighted the salient difficulties of cross-cultural psychiatry, specifically the translation of materials to local languages in many low and middle-income countries. However, because it is common to observe evolution in the terms that describe mental and emotional experiences, this situation is likely to improve over the years as mental health awareness increases. The translation of the guide into Kiswahili may therefore improve in the long term.

### Pilot testing of adapted guide and feedback workshop with the national and county government as well as implementation of suggested changes

Successful pilot testing of the guide among the healthcare providers indicated that the guide can be used in coastal Kenya for diagnosis and management of common mental disorders. Like in other settings (Akol *et al*., 2017, Robles *et al*., 2020), there was an improvement in the post-test scores in most of the modules except for the epilepsy module. An improvement in the post-training scores of the adapted guide showed that the guide improved the level of knowledge of the healthcare providers about diagnosis and management of mental and substance use disorders. There was no improvement in the epilepsy module perhaps because the study population was involved in epilepsy research studies (Mbuba and Newton, 2009; Ibinda *et al*., 2014; Kariuki *et al*., 2015)and had higher baseline levels of knowledge about the disorder as shown by the high pre-training scores. Feedback from the specialists provided useful insights on the use of a step-wise approach in training healthcare providers based on their qualifications and level of experience.

Although the specialists recommended the exclusion of child and adolescent and dementia modules from the final version, evidence that emerged after the adaptation process showed that the burden of child and adolescent mental and behavioural problems is high (Kariuki *et al*., 2017) and this could be applicable to dementia. Therefore, future implementation plans will consider this new evidence and will include adaptation of these module. This will provide a unique opportunity for the evaluation of the implementation of these modules, especially in sub-Saharan Africa where very few studies have implemented this module (Akol *et al*., 2017).

### Study limitations

We did not adapt the psychological interventions' components of the guide for use by lay health workers therefore patients requiring specialised services such as cognitive behavioural therapy, will still need to be referred to specialists. However, at present, there is a lot of effort by the government (GOK, 2019) to improve mental healthcare in the country and we anticipate that the human resource for mental health is likely to improve including increase in the number of specialists capable of providing specialised psychological interventions such as cognitive behavioural therapy. Additionally, to utilise the robust community strategy of provision of health services in Kilifi County, the study team plans to pilot psychological interventions for lay health workers, that are currently under development.

The pilot did not evaluate knowledge retention among primary healthcare workers after training. However, implementation of the guide, which will be facilitated by KWTRP, will include close monitoring and supervision by master trainers who will be trained healthcare providers within the County.

## Conclusions

The process of contextualising the mhGAP-IG in coastal Kenya involved extensive planning and consultation at multiple levels of government with numerous stakeholders. The key findings from the process included the challenges of different levels of experience in MNSD among healthcare staff , difficulties in translation due to lack of words in Kiswahili which is the local language and policy limitations which affected supply of drugs and availability of specialised human resource. Successful pilot testing of the adapted guide provided a basis for large-scale implementation of the mhGAP-IG in coastal Kenya, which is currently in the planning phases. However, the process demonstrated the need to continue engagements with policy makers to improve resource allocation towards mental health and sustain the implementation efforts. Implementation will also require enough resources to provide sustained training and supervisory support to the primary healthcare providers for successful outcomes. Lastly, barriers to access of care such as stigma and other social determinants of mental health were not addressed during the consultative meetings and these may hinder the uptake of services during the implementation process. Participants of the consultative process may have excluded these barriers in the discussions due to social desirability associated with the use of qualitative methods of data collection. However, the research team from KEMRI Wellcome Trust is currently conducting research to provide baseline data on the levels of stigma among people with MNSD.
